# Testicular orphan nuclear receptor 4-associated protein 16 promotes non-small cell lung carcinoma by activating estrogen receptor β and blocking testicular orphan nuclear receptor 2

**DOI:** 10.3892/or.2012.2107

**Published:** 2012-10-26

**Authors:** FANG FANG, QINGFENG ZHENG, JIANZHI ZHANG, BIN DONG, SAINAN ZHU, XIAOYUN HUANG, YANG WANG, BINGTIAN ZHAO, SHAOLEI LI, HONGCHAO XIONG, JINFENG CHEN, NAN WU, SONYA WEI SONG, CHAWNSHANG CHANG, YUE YANG

**Affiliations:** 1 Department of Thoracic Surgery II, Peking University Cancer Hospital and Institute, Beijing 100142; 2 Department of Pathology, Peking University Cancer Hospital and Institute, Beijing 100142; 3 Clinical Research Laboratory, Key Laboratory of Carcinogenesis and Translational Research (Ministry of Education), Peking University Cancer Hospital and Institute, Beijing 100142; 4 Department of Medical Statistics, Peking University First Hospital, Beijing 100034, P.R. China; 5 Departments of Pathology, Urology, Radiation Oncology, and Cancer Center, George Whipple Laboratory for Cancer Research, University of Rochester Medical Center, Rochester, NY 14642, USA

**Keywords:** ERβ, testicular orphan nuclear receptor 2, TR4-associated protein 16, non-small cell lung cancer

## Abstract

The possible involvement of estrogen receptors (ERs) and testicular orphan nuclear receptors (TRs) in human non-small cell lung carcinoma (NSCLC) has been suggested, but their precise roles and their relationship remain largely unknown. This study aimed to investigate whether TR4-associated protein 16 (TRA16) regulates the ERβ and TR2 pathways and could be a potential target in NSCLC. We used tissue microarrays including NSCLC tissues (n=154) and negative controls (n=14) to examine the expression of TRA16 and ERβ, and *in vitro* reporter gene assays, the mammalian two-hybrid method and immunoprecipitation in Cos-1 cells to investigate the relationships among TRA16, ERβ and TR2. We found that TRA16 was highly expressed in approximately 90% of the NSCLC tissues examined. TRA16 overexpression was significantly associated with TNM stage, tumor size, lymph node metastasis, tumor thrombus in vein, tumor differentiation and prognosis of NSCLC patients, in which TRA16 was shown to be an independent prognostic factor. Introduction of TRA16 into Cos-1 cells enhanced cell proliferation. Co-expression of TRA16 and ERβ in Cos-1 cells using different reporter gene systems and mammalian two-hybrid approaches revealed that TRA16 enhanced ERβ-mediated transcriptional activity. By adopting similar approaches, and immunoprecipitation and immunocytofluorescence assays, we found that TRA16 also interacted with TR2, and blocked the TR2 inhibitory effect on ERβ. Our findings demonstrate that TRA16 could be a promising diagnostic and prognostic biomarker in NSCLC, and promotes cancer cell growth through activation of the ERβ pathway by interacting with ERβ and TR2.

## Introduction

Lung cancer is the leading cause of cancer-related death throughout the world (1). Since the lack of symptoms and little help from traditional X-rays on early stage lung cancer, most patients are diagnosed with advanced stage disease and thus have poor prognosis. Therefore, there is an urgent need to explore new biomarkers to diagnose and therapeutic strategies to treat this disease at its early stages.

Previous studies showed that a great majority of NSCLC highly expressed estrogen receptor beta (ERβ) (2–6). Human normal bronchial and alveolar epithelial cells were also reported to express ERβ, which was considered to contribute to the maintenance of normal lung tissue (7). Niikawa *et al* first reported that intratumoral estradiol (an activator of ERβ) concentration was significantly higher than that of corresponding non-neoplastic lung tissues, and positively associated with tumor size and Ki-67 labeling index (LI) in ER-positive NSCLC tissues (8). Aromatase (estrogen synthetase) expression was also detected in NSCLC tissues (9,10). It was reported that activated ERβ signaling promoted carcinogenisis through stimulating abnormal cell proliferation (8,11).

Steroid hormones play important physiological roles in cell differentiation, development, and homeostasis through their binding to specific receptors of the nuclear receptor superfamily (12–16). Some studies showed that human testicular orphan nuclear receptor-4 (TR4) repressed the ERβ-mediated transactivation of its downstream signal pathways in lung cancer cells and indicated that TR4 might be a potential tumor suppressor gene (17). We previously found that TR4-associated protein (TRA16) suppressed the TR4-mediated ERβ activity and its downstream signaling by interrupting binding of TR4 with TR4 response element (TR4RE) and blocking TR4 dimerization in lung cancer cells (18), suggesting that TRA16 plays a role in lung cancer development.

TR2 is highly homologous with TR4 and both were reported to act as transcriptional factors to regulate gene expression in embryonic stem cells and early embryos (19). TR2 was also shown to repress the ERβ-mediated transactivation of its downstream signal pathway in lung cancer cells (20). Thus, this study further investigated the role of TRA16 in NSCLC and the relationships among TRA16, TR2 and ERβ. We demonstrate that TRA16 is highly expressed in NSCLC tissues, and its expression level is increased with increased malignancy and negatively correlated with survival of NSCLC patients. Furthermore, we show that TRA16 promotes cancer cell growth through activating ERβ and blocking TR2 activity.

## Materials and methods

### Patients, tissue specimens, and cell lines

This study was approved by Institutional Review Committee of Beijing Cancer Hospital. As summarized in Table I, a total of 154 NSCLC specimens were obtained from patients who underwent surgical resection from 1995 to 2000 in the Department of Thoracic Surgery II of Beijing Cancer Hospital. The median overall survival time was obtained from all the patients examined and the follow-up was from September 1995 till September 2005. The median follow-up time was 38.9 months (from 1.5 to 109 months). Lung tissues from 12 benign lung disease patients and 2 thoracic injury patients were collected as controls. All the patients and controls gave informed consent for the study.

NSCLC cell types included squamous carcinoma, adenocarcinoma (including bronchioloalveolar carcinoma), large cell carcinoma, adenosqumous carcinoma, and salivary adenocarcinoma. Benign diseases included inflammatory pseudotumor, tuberculosis, sclerotic hemangioma, and harmatoma. The tissue samples were histologically confirmed by two independent pathologists. Monkey kidney cell line COS-1 was obtained from the American Type Culture Collection and maintained in DMEM with 10% fetal bovine serum (FBS) (Gibco).

### Tissue microarray and immunohistochemical (IHC) analysis

Tissue microarray was constructed as described previously using a manual tissue arrayer (Beecher Instruments) (21). Briefly, H&E slides of each tissue sample were reviewed by two pathologists. A tumor tissue block was chosen for the tissue microarray. Scarred, myxoid, and hypocellular areas were avoided. Two tissue cores with a diameter of 0.6-mm were taken from each block and inserted into the blank tissue microarray block. The whole set of tissue array contained two chips (A and B), in which chip A had 78 NSCLCs and 3 benign controls and chip B contained 76 NSCLCs and 11 benign controls.

The tissue microarrays were deparaffinized in xylene and ethanol. Antigen retrieval was done by heating the slides in an autoclave at 120°C for 3 min in citric acid buffer. The primary antibody was mouse monoclonal anti-TRA16 (22) (1:200 dilution in PBS) or rabbit monoclonal anti-ERβ (1:100 dilution in PBS, Dako). The immunostaining was carried out using the EnVision method (Dako) according to the manufacturer’s instructions. Brown staining was considered positive. Normal mouse IgG was used instead of the primary antibody as a negative control.

### Stable transfection of Cos-1 cells and methyl thiazolyl tetrazolium (MTT) assay

Cos-1 cells, which are TR2-negative and express very low level of TRA16, were transfected with pBig or pBig-TRA16 using SuperFect (Qiagen). The cells were then selected using 100 μg/ml hygromycin B to generate stable clones, which were confirmed by reporter gene assay. The established stable pBig-TRA16-Cos-1 cells (5×10^4^) per well were seeded in 12-well plates. After 12 h, the medium was changed to DMEM medium with 10% FBS for another 4 days and then treated with doxycycline (6 μg/ml) or DMSO for induction of TRA16 expression for 24 h.

For the MTT assay, 2×10^3^ of Cos-1 cells per well were plated in 96-well plates with RPMI-1640 medium containing 10% FBS and incubated at 37°C overnight. After 1, 2, 3, or 4 days, 200 μl of MTT (5 mg/ml, Sigma) was added to each well for 3-h incubation, then 2 ml of 0.04 M HCl in isopropyl alcohol was added to each well to stop the reaction. After 5 min of incubation at room temperature, the absorbance was read at 570 nm.

### Transient transfection and mammalian two hybrid assays

Cos-1 cells (2.5×10^4^) per well were plated in 12-well plates. The next day, the medium was changed to a medium containing 10% charcoal-stripped serum to deprive the cells of steroid hormones. The cells were then transfected the following day. Transfections were performed using SuperFect (Qiagen) according to the manufacturer's instructions. The cells were transiently cotransfected with reporter plasmids pERE-TK-Luc (a gift from Dr M. Nichols at University of Pittsburgh, Pittsburgh, PA) and ERβ with or without TRA16. After 24 h, the cells were incubated with 10 nM estrogen or androgen if necessary for 24 h and then harvested using lysis buffer (Promega) to detect the interaction between TRA16 and ERβ.

To further prove the interaction between TRA16 and ERβ, the Cos-1 cells were transiently co-transfected with reporter plasmid pG5-Luc and each of GAL4DBD, VP16, VP16-TRA16, and GAL4-ERβ in mammalian two hybrid system. As negative controls, the Cos-1 cells were transiently cotransfected with PPRE-Luc, MMTV-Luc, PSA-Luc, ARE-Luc, and the pRL-CMV (Promega, Madison, WI) to confirm the specifity of the interaction between TRA16 and ERβ.

To assess the interaction between TRA16 and TR2, we adopted three approaches. First, we transiently co-transfected TR2 and TRA16 or TR2 alone into Cos-1 cells to measure the inhibition of TR2 transcriptional activity by TRA16. Second, pBig-TRA16-Cos-1 and pBig-Cos-1 stable cells were transiently co-transfected with HCR-1-Luc reporter and TR2 for 24 h, then transfected cells were treated with doxycycline to induce TRA16 expression, and measure the TR2 activity. Third, we transiently co-transfected the reporter plasmid pG5-Luc and each of GAL4DBD, VP16, VP16-TR2, and GAL4-TRA16 in mammalian two-hybrid system to further prove the interaction between TRA16 and TR2. The luciferase activity was measured using the Dual-Luciferase System (Promega) by TD 20/20 luminometer. Values of the luciferase activity were corrected for protein concentration and presented as the mean ± SD of three independent experiments.

### Immunoprecipitation assay

Cos-1 cells were transiently transfected with TR2 and either TRA16 or pcDNA4 control for 48 h and then harvested and dissolved in lysis buffer (1% Nonidet P-40, 10% glycerol, 135 mM NaCl, 40 mM Tris, pH 7.4, 1 mM phenylmethylsulfonyl fluoride, 1 mM dithiothreitol, and 1X protease inhibitor cocktail Roche). Cell lysates containing 500 μg of proteins were precleared with 20 μl of protein A/G Plus-agarose and 1.0 μg of normal mouse IgG (Santa Cruz Biotechnology) for 30 min. The supernatant was then mixed with a 1:100 dilution of mouse TR2 antibody at 4°C for 2 h, followed by adding protein A/G Plus-agarose and incubated for another 2 h. Immunoprecipitates obtained by spinning down protein A/G Plus-agarose were washed with PBS for three times and separated on SDS-8% PAGE. After transferring to the membrane, the protein was detected by anti-His-tag or anti-TR2 antibodies.

### Immunocytofluorescence assay

Cos-1 cells transiently co-transfected with TR2 and TRA16 were seeded on two-well Lab Tek Chamber slides (Nalge) for 48 h. Immunostaining was performed as described previously (18) by incubating with mouse anti-TRA16 monoclonal antibody and/or rabbit anti-TR2 monoclonal antibody, and then followed by incubating with either fluorescein-conjugated goat anti-mouse or anti-rabbit antibodies. Coverslips were fixed on the glass slides with a drop of DAPI to stain the nucleus. The slides were observed under 400-fold magnification of a fluorescence microscope or confocal fluorescence microscope.

### Data analysis

SPSS v11.0 software was used to perform statistical analysis. Chi-square test was used to analyze the assocation between TRA16 expression and different clinical factors in NSCLC patients. Univariate and multivariate Cox regression hazards models were used to analyze the correlation of individual factors with overall survival of NSCLC patients. Log-rank test and Kaplan-Meier survival analysis were used to examine the effect of individual factors on prognosis of NSCLC patients. Values of P<0.05 were considered significant.

## Results

### TRA16 is highly expressed in non-small cell lung cancer

**Figure 1:**
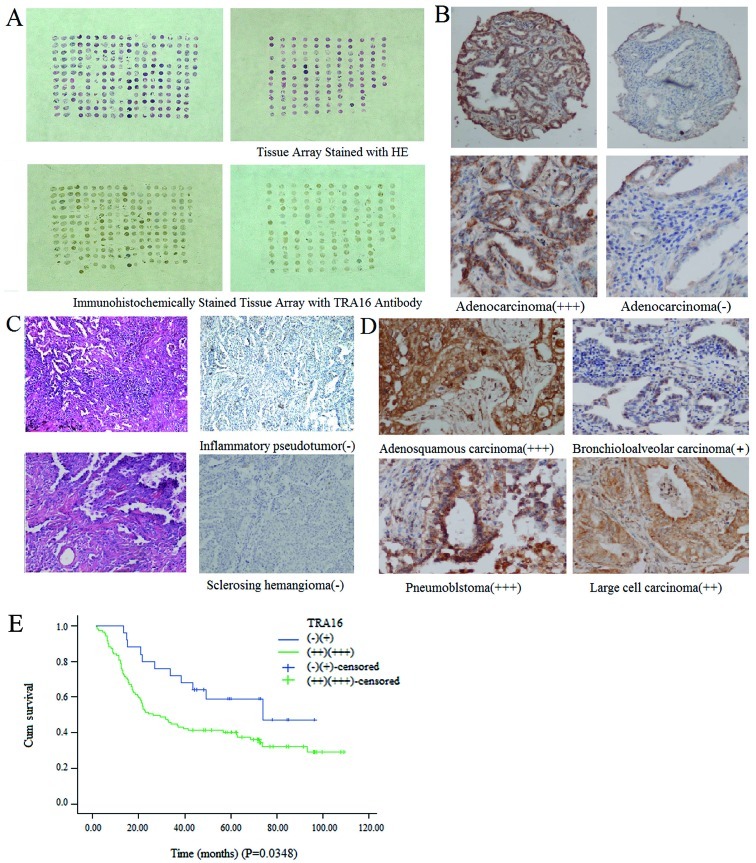
TRA16 expression in NSCLC and control tissues by tissue microarray analysis and its association with overall survival (OS) in NSCLC patients. (A) Two tissue chips were stained with H&E (upper panel) and TRA16 antibody (lower panel). Among total 154 cases, 134 cases were qualified to be analyzed. (B) Examples of TRA16 expression in two adenocarcinoma cases. Case 37151 was TRA16-positive (+++), and Case 35983 was TRA16-negative (−). (C) TRA16 staining in different benign lung diseases showing TRA16-negative (−). (D) TRA16 expression in different cell types of NSCLC. (E) The Kaplan-Meier survival analysis and a log-rank test showed that lung cancer patients with high TRA16 expression had a significantly worse OS (A, P=0.0348).

We previously found that TRA16 was highly expressed in lung adenocarcinoma cell line H1299 (18). To investigate the expression of TRA16 in NSCLC tissues, we performed tissue microarrays with 154 NSCLCs, in which 134 NSCLC samples were qualified for data analysis (Table II). The results of TRA16 tissue microarray and its expression level and distribution in different NSCLC cell types are exemplified in Fig. 1A-D. We found that all the control samples were TRA16-negative, but 120 lung cancer samples were TRA16-positive (+, ++, or +++) with the positive rate of 89.55% (120/134) (P<0.001). When categorizing the samples with the staining intensity of ++ or +++ as strong positive, there were 105 strong positive samples accounting for 78.36% (105/143) of the NSCLCs. The 105 samples included 55 adenocarcinoma (83.3%, 55/66), 43 squamous carcinoma (71.7%, 43/60). No correlation of TRA16 expression with NSCLC cell types was observed (P=0.230). The Chi-square test indicated that the TRA16 expression was correlated with TNM stage (P=0.016).

### TRA16 expression is correlated with prognosis of NSCLC patients

An univariate Cox regression analysis of clinicopathologic parameters and TRA16 expression showed that TNM stage, tumor size, lymph node metastasis, tumor thrombus, cell differentiation, and TRA16 expression were significantly associated with overall survival of NSCLC patients (Table III). The multivariate Cox regression analysis showed that lymph node metastasis, tumor thrombus formation, poor cell differentiation, and high expression of TRA16 were independent prognostic factors in NSCLC (Table III). The Kaplan-Meier analysis showed that the patients with higher TRA16 expression had significantly shorter overall survival than those with lower TRA16 expression (P=0.0348) (Fig. 1E), similarly to the standard clinical prognostic factors of TNM stage, lymph node metastasis, and tumor differentiation.

### TRA16 expression is correlated with ERβ expression in NSCLC tissues

Our previous studies demonstrated that TRA16 inhibited the function of TR4 in lung cancer cell line H1299 and resultantly relieved TR4-repressed ERβ activity (18). To further determine the relationship between TRA16 and ERβ in NSCLC, we analyzed the expressions of TRA16 and ERβ in 71 NSCLC tissues and 2 benign samples using the tissue microarrays. We found that among the 71 NSCLC tumors, 35 cases were ERβ-positive with 22 cases (+), 9 cases (++), and 4 cases (+++), while 36 cases and two benign controls were ERβ-negative. When using the TRA16 expression to stratify the 71 cases into no or low (+) level group (n=15) and high level (++ − +++) group (n=56) and then analyzing the difference of the ERβ expression between the two groups, we found that the ERβ expression in the high level group of TRA16 was significantly higher than that in the no or low level group, while only one of 15 samples in the no or low level group of TRA16 showed ERβ-positive (P<0.001) (Table II), suggesting their functional association.

### TRA16 enhances cell proliferation in Cos-1 cells

The increased expression of TRA16 in NSCLC indicated that TRA16 could play an important role in lung cancer development. Thus, we performed the MTT assay to study the effect of TRA16 on cell growth by transfecting doxycycline-inducible TRA16 in pBig vector into Cos-1 cells. We found that the doxycycline-treated group had a significantly higher cell growth than the DMSO-treated control group.

### Interaction of TRA16 and ERβ enhances ERβ activity

**Figure 2:**
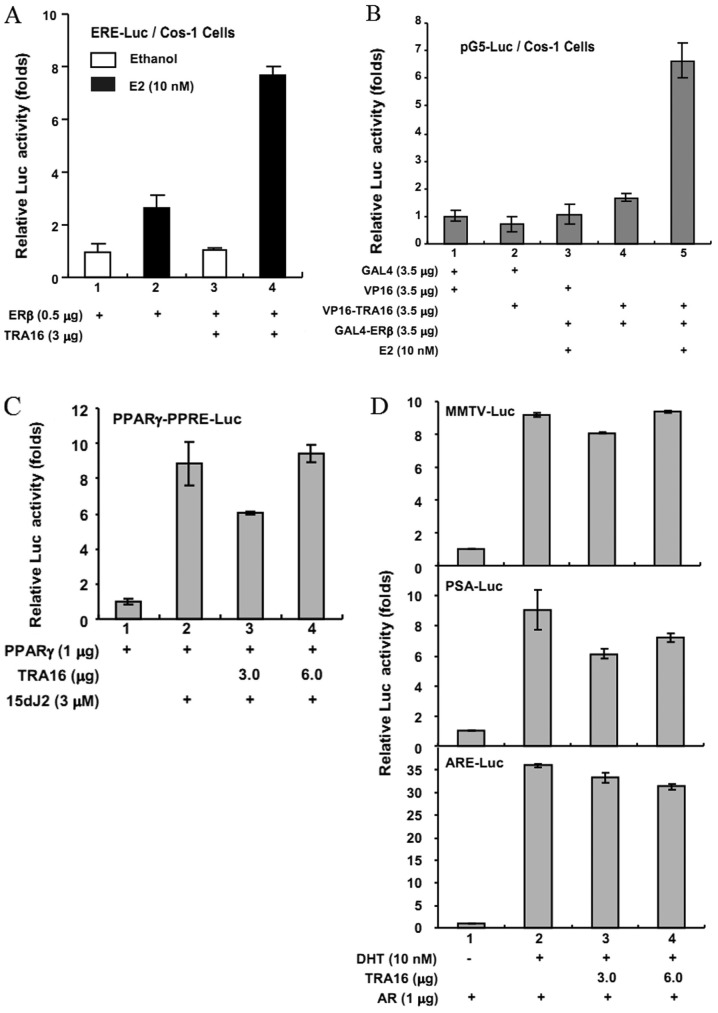
The interaction of TRA16 and ERβ on the ERβ activity in mammalian Cos-1 cells. (A) Cos-1 cells were transiently co-transfected with 3 μg of ERE-Luc reporter, 0.5 μg of ERβ, and 3 μg of TRA16 expression plasmids for 24 h, then treated with either 10 nM E_2_ or ethanol vehicle for the other 24 h to measure the luciferase activities. (B) Cos-1 cells were transiently co-transfected with 3 μg of reporter plasmid pG5-Luc and 3.5 μg each of GAL4DBD, VP16, VP16-TRA16, and GAL4-ERβ in various combinations, then treated with either 10 nM E_2_ or ethanol vehicle for 24 h to measure luciferase activities. (C) Cos-1 cells were transiently co-transfected with 3 μg of reporter plasmid PPRE-LUC, 1 μg of PPARγ, and different doses (3.0 and 6.0 μg) of TRA16, then treated with 3 μM of 15dJ2 (an activator of PPARγ) for another 24 h to measure luciferase activities. (D) Cos-1 cells were transiently co-transfected with 3 μg of different reporter plasmids (MMTV-Luc, PSA-Luc, or ARE-Luc), and both AR and TRA16 (ratio: 1:3 and 1:6) for 24 h, and then treated with 10 nM of DHT to measure luciferase activities.

To further explore the functional association between TRA16 and ERβ, we performed several reporter gene assays by transient transfection of different combinations of plasmids into Cos-1 cells (Fig. 2). We found that when transiently co-transfecting ERβ response element (ERE)-Luc reporter and ERβ with or without TRA16 into Cos-1 cells, after adding 10 nM of estradiol (E_2_) to stimulate ERβ activity, TRA16 significantly enhanced the ERβ activity in Cos-1 cells compared with the ERβ alone (Fig. 2A). Further, in the mammalian two-hybrid assay by transfecting the reporter plasmid pG5-Luc and each of GAL4DBD, VP16, VP16-TRA16, and GAL4-ERβ in various combinations as indicated into Cos-1 cells, we found that after the stimulation of E_2_, the co-presence of TRA16 and ERβ significantly enhanced the ERβ activity in Cos-1 cells (Fig. 2B), indicating that TRA16 interacted with ERβ.

To clarify the specificity of the association of TRA16 and ERβ, similar but negative control experiments were performed by co-transfecting TRA16 and PPAR or androgen receptor (AR) in Cos-1 cells. As shown in Fig. 2C and D, TRA16 had little effect on activation of both PPAR and AR.

### TRA16 interacts with TR2 enhancing ERβ activity

**Figure 3:**
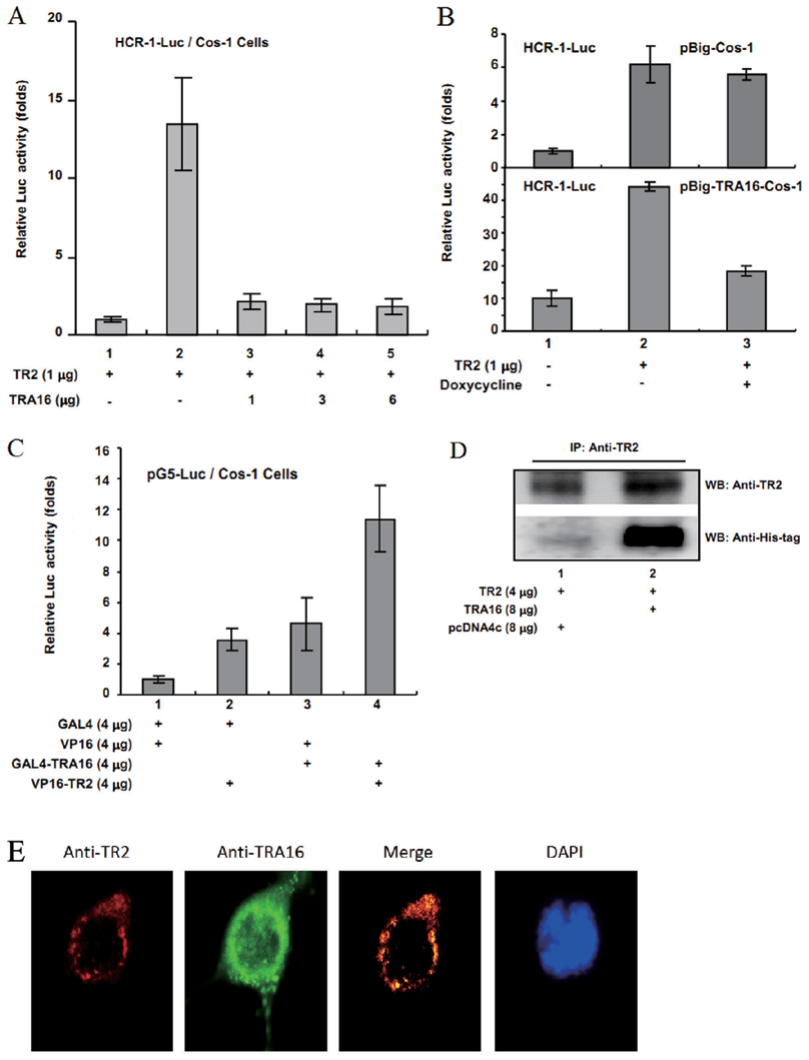
Interaction between TRA16 and TR2 detected by different methods. (A) Cos-1 cells were transiently co-transfected with 3 μg of HCR-1-Luc reporter, 1 μg of TR2, and increased amount of TRA16 expression plasmid, and 24 h after transfection, the luciferase activity was measured. (B) pBig-TRA16-Cos-1 and pBig-Cos-1 stable cells were transiently co-transfected with 3 μg of HCR-1-Luc reporter, and 1 μg of TR2 for 24 h, then treated with either 6 μg/ml doxycycline or DMSO vehicle for another 24 h, and the luciferase activity was measured. (C) Cos-1 cells were transiently co-transfected with 4 μg of reporter plasmid pG5-Luc and 4 μg each of GAL4DBD, VP16, VP16-TR2, and GAL4-TRA16 in various combinations as indicated, and then cultured for 24 h to measure the luciferase activities. (D) Cos-1 cells were transiently co-transfected with 4 μg TR2 and 8 μg either TRA16 or pcDNA4c for 48 h, then the cell lysates were harvested, precleared and added with anti-TR2 antibody and protein G-agarose beads. The immunoprecipitates obtained were subjected to 8% SDS-PAGE to detect the pull-down protein. (E) Cos-1 cells were transiently co-transfected with TR2 and TRA16, then stained with anti-TRA16 or anti-TR2 antibody, and then corresponding second antibodies. DAPI was added to stain the nucleus. The cells were then observed under the fluorescent microscopy. The green signal represents TRA16, the red signal represents TR2, the yellow signal represents co-localization of TRA16 and TR2, and the blue signal represents the DAPI-stained nucleus.

We previously demonstrated that TRA16 bound to TR4 and blocked TR4 dimerization and thus inhibited TR4 activity in lung cancer cells (18). TR2 and TR4 are highly homologous and TR2 can suppress ERβ-mediated cell growth in lung cancer cells *in vitro*(19,20). To assess a potential relationship between TRA16 and TR2, we performed similar reporter gene assays as described above. We found that when HCR-1-Luc reporter gene and TR2 with or without TRA16 were co-transfected into Cos-1 cells, TRA16 strongly inhibited TR2 transcriptional activity in Cos-1 cells (Fig. 3A). Similarly, the doxycycline-inducible assay using the pBig-Cos-1 and pBig-TRA16-Cos-1 cell system showed that after treatment of 6 μg/ml doxycycline, TR2 activity was significantly lower in pBig-TRA16-Cos-1 cells than that in pBig-Cos-1 cells (Fig. 3B). Moreover, the mammalian two-hybrid assay demonstrated that the co-expression of TRA16 and TR2 significantly enhanced the reporter Luc activity compared with TR2 or TRA16 alone (Fig. 3C), suggesting the association of TRA16 and TR2.

Furthermore, the immunoprecipitation assay showed that TRA16 directly interacted with TR2 (Fig. 3D), and TRA16 and TR2 were co-localized in the nuclear membrane by immunocytofluorescence assay (Fig. 3E). Taken together, the findings suggest that TRA16 interact with TR2 to release TR2-inhbitory role in ERβ activity in addition to its direct stimulation of ERβ signaling.

## Discussion

Our study reports that TRA16 is highly expressed in NSCLCs and its expression level is negatively correlated with the overall survival of NSCLC patients, and it enhances ERβ signaling pathway by direct stimulation of ERβ activity and through suppressing TR2-inhibitory effect on ERβ for cancer cell growth, suggesting TRA16 is a promising diagnostic and prognostic biomarker and potential target in NSCLC.

Tumor biomarkers, such as CEA, CA125, NSE, CYFRA21-1, and p53, are currently used in cancer diagnosis and prognosis, but few of them are ideal for NSCLC because of low sensitivity and specificity (23–25). For stage I NSCLC patients, the five-year survival rate exceeds 60%, but the patients with metastatic spread to regional or distant sites, who account for at least three quarters of lung cancer patients at the time of diagnosis, have only approximately 15% five-year survival rate (26). Therefore, if the NSCLC can be diagnosed in early stages, the patient survival rate and time could be significantly improved. This study demonstrates that TRA16 is highly expressed in approximately 90% of NSCLCs, in which over 71% of stage I NSCLC overexpress TRA16, which is not expressed in lung benign diseases and normal lung tissues, indicating its potential for NSCLC diagnosis, especially for early stage NSCLC.

Furthermore, our findings of the correlation of increased expression of TRA16 with increased malignancy of NSCLC demonstrate that TRA16 could be a valuable biomarker to monitor the progression of the tumor, and provide physicians with useful information to evaluate the severity of the disease and to choose proper treatments for NSCLC patients.

For a long time, researchers have proposed that sexual hormone, such as progesterone and estrogen, may play important roles in lung cancer carcinogenesis and development (27,28), which was supported by recent clinical findings of abnormal expression of ERβ (2–6) and increased intratumoral estrodial concentration in NSCLC tissues (8). There are several possible mechanisms that may be involved in the abnormal activation of hormone related signaling transduction pathways. First, the high serum hormone level or high hormone receptor expression level can up-regulate downstream signaling pathways to stimulate target cell proliferation (8). Second, the mutation of hormone receptors may increase the ligand binding affinity or be activated by other ligands (29,30). Third, the abnormality of hormone receptor co-factors can active the downstream signaling transduction pathway, and then promote oncogenesis (31).

TRA16 belongs to the co-factors of nuclear receptors (NRs) with which it interacts to affect the transcriptional activity of NRs. Our findings that the concordant expressions of TRA16 and ERβ in NSCLC and their interaction suggest that TRA16 may serve as an upstream positive regulator of ERβ in NSCLC. Moreover, we have observed the interaction between TRA16 and TR2. Therefore, possible interactions among TRA16, TR2, TR4, and ERβ could be proposed. TR2 and TR4 inhibit ERβ signaling pathways. In addition to its direct stimulation of ERβ, TRA16 also directly interacts with TR2 and TR4 that results in the release of ERβ from TR2 and TR4 inhibition, which consequently leads to the tumorigenesis. Since it is highly and specifically expressed in tumor tissues, TRA16 may be used as a tumor-specific therapeutic target for lung cancer treatments in the form of anti-TRA16 antibody or TRA16 shRNA.

Therefore, there are still many questions to be answered, such as what are the upstream regulators of TRA16 and its interaction sites with ERβ and TR2, and whether the TRA16 expression can be detected in circulating cancer cells in patient blood that could be used to easily screen and diagnose patients with NSCLC, all of which will be investigated in future studies.

## Figures and Tables

**Table I:** Clinicopathological characteristics of patients.

Characteristic	No. of patients (%)
Gender	
Male	113 (73)
Female	41 (27)
Age	
≤60	68 (44)
>60	86 (56)
Histology	
Squamous carcinoma	73 (47)
Adenocarcinoma	70 (45)
Others	11 (7)
TNM stage	
I	54 (35)
II	54 (35)
IIIA	46 (30)
Tumor size	
T1	18 (12)
T2	62 (40)
T3	74 (48)
Lymph node metastasis	
No	105 (68)
Yes	49 (32)
Tumor thrombus	
No	131 (85)
Yes	23 (15)
Differentiation	
Poor	66 (43)
Moderate	46 (30)
High	39 (25)

**Table II:** The expression level of TRA16 in human lung clinical samples.

TRA16 expression	++/+++ (%)	−/+	Total	P
Control	0 (0)	14	14	<0.001
Benign disease	0 (0)	12	12	
Inflammatory pseudotumor	0 (0)	4	4	
Tuberculosis	0 (0)	4	4	
Sclerotic hemangioma	0 (0)	2	2	
Harmatoma	0 (0)	2	2	
Normal	0 (0)	2	2	
Lung cancer cell type	105 (78.36)	29	134	0.230
Squmous cell	43 (71.67)	17	60	
Adenocarcinoma	55 (83.33)	11	66	
Others	7 (87.50)	1	8	
TNM stage	128 (83.12)	26	154	**0.016**
I	39 (72.22)	15	54	
II	46 (85.18)	8	54	
IIIA	43 (93.48)	3	46	
ER expression	56 (78.87)	15	71	**<0.001**
++/+++	34 (97.14)	1	35	
−/+	22 (61.11)	14	36	

**Table III:** Univariate and multivariate Cox regression analysis of clinicopathological and genetic factors for prognosis in NSCLC patients.

Clinicopathological and genetic factors	Patient no.	Median OS (month)	P-value	

			Univariate analysis	Multivariate analysis
Gender			0.2372	
Male	113	37		
Female	41	63		
Age			0.8500	
≤60	68	39		
>60	86	34		
Smoking history			0.7769	
No	92	37		
Yes	62	39		
Histopathological classification			0.6300	
Squamous cell	73	38		
Adenocacinona	70	37		
Others	11	41		
TNM stage			**0.0000**	
I	54	93		
II	54	42		
IIIA	46	17		
Tumor size			**0.0093**	
T1	18	93		
T2	62	63		
T3	74	23		
Lymph node metastasis			**0.0000**	**0.007**
No	105	73		
Yes	49	21		
Tumor thrombus in vana			**0.0177**	**0.007**
No	131	49		
Yes	23	21		
Cell differentiation			**0.0151**	**0.000**
Well-differentiated	39	93		
Moderately-differentiated	46	42		
Poorly-differentiated	66	22		
TRA16 expression			**0.0348**	**0.020**
(−) (+)	25	65		
(++) (+++)	109	26		

OS, overall survival.

## Abbreviations

ERβ: estrogen receptor β
TR2: testicular orphan nuclear receptor 2
TRA16: TR4-associated protein 16
NSCLC: non-small cell lung cancer
OS: overall survival
E2: estradiol
NRs: nuclear receptors
LI: labeling index
TR4RE: TR4 response element
FBS: fetal bovine serum
MTT: methyl thiazolyl tetrazolium
